# Relating Cortical Atrophy in Temporal Lobe Epilepsy with Graph Diffusion-Based Network Models

**DOI:** 10.1371/journal.pcbi.1004564

**Published:** 2015-10-29

**Authors:** Farras Abdelnour, Susanne Mueller, Ashish Raj

**Affiliations:** 1 Radiology, Weill Cornell Medical College, New York, New York, United States of America; 2 Radiology, University of California San Francisco, San Francisco, California, United States of America; Indiana University, UNITED STATES

## Abstract

Mesial temporal lobe epilepsy (TLE) is characterized by stereotyped origination and spread pattern of epileptogenic activity, which is reflected in stereotyped topographic distribution of neuronal atrophy on magnetic resonance imaging (MRI). Both epileptogenic activity and atrophy spread appear to follow white matter connections. We model the networked spread of activity and atrophy in TLE from first principles via two simple first order network diffusion models. Atrophy distribution is modeled as a simple consequence of the propagation of epileptogenic activity in one model, and as a progressive degenerative process in the other. We show that the network models closely reproduce the regional volumetric gray matter atrophy distribution of two epilepsy cohorts: 29 TLE subjects with medial temporal sclerosis (TLE-MTS), and 50 TLE subjects with normal appearance on MRI (TLE-no). Statistical validation at the group level suggests high correlation with measured atrophy (*R* = 0.586 for TLE-MTS, *R* = 0.283 for TLE-no). We conclude that atrophy spread model out-performs the hyperactivity spread model. These results pave the way for future clinical application of the proposed model on individual patients, including estimating future spread of atrophy, identification of seizure onset zones and surgical planning.

## Author Summary

Medial temporal lobe epilepsy is the most common form of focal epilepsy. In this work we investigate two models describing the dynamics of epilepsy. In the first model the extrahippocampal spread of seizure activity is primarily responsible for the apparent topographic distribution of atrophy. The second hypothesis is that loss of hippocampal neurons leads to remote deafferentation followed by gradual and progressive neuronal loss in connected regions. Impoverishment of hippocampal connections can lead to reduced complexity of remote circuitry. The purpose of this work is to develop network theoretic models of regional atrophy dynamics resulting from each of the above hypotheses, and to statistically determine which model is a better descriptor of the spatial patterning of real TLE atrophy. Both models are based on simple graph theoretic models of influence spread as a network diffusion process, enacted on the brains structural connectivity network.

## Introduction

Mesial temporal lobe epilepsy (TLE) is the most common form of focal epilepsy, and is characterized by seizure focus in the mesial temporal lobe from where it can spread into other neocortical regions. Based on their aspect on the structural MRI and histopathology, two subtypes of TLE are distinguished. One that is characterized by prominent hippocampal atrophy or mesial-temporal sclerosis (TLE-MTS) and one where the hippocampus appears completely normal (TLE-no). In both cases, new morphometric analysis using MRI shows consistent evidence of extra-hippocampal and extratemporal atrophy [1–6]. It is shown by Coan *et al* that atrophy progression in temporal lobe epilepsy depends on lateralization [7]. In a related work, it is observed that patients with TLE show progressive neocortical damage, likely a result of seizures [8]. Atrophy distributions appear to vary significantly, and both widespread [9] and restricted distribution of gray matter (GM) atrophy [4, 10] have been observed in TLE subjects. In most cases however, damaged regions tend to be functionally and anatomically connected to the hippocampus and other medial temporal structures [3, 11–13].

Thus, both epileptogenic activity and gross atrophy in epilepsy establish themselves at networked sites, and appear to spread along fiber pathways. However, it is not clear whether activity or atrophy is the propagating event, and two hypotheses have been proposed. First, seizure activity is the primary propagating quantity, and neuronal damage results from excitotoxicity [14]. In this view, the extrahippocampal spread of seizure activity is primarily responsible for the apparent topographic distribution of atrophy [11, 13, 15]. Although atrophy in all regions might worsen with time, there is no progressive outward spread of atrophy. The medial temporal and limbic structures have important connections to each other and to the hippocampus; these connections provide significant feedback mechanisms [16], leading to the spread of initially local epileptogenic activity to widespread connected regions. The process of neuronal death secondary to sustained hyperactivity is not fully understood, but likely involves mutual inter-cortical trophic exchanges, ultimately leading to long-lasting remodeling of brain networks [17]. Once epileptogenic activity is established in a region, over time it causes local atrophy via a complex cascade of neurobiological events [18], a process called excitotoxicity. The close correspondence between the sites of epileptogenic activity and atrophy patterns observed in TLE patients described above supports this hypothesis. The second hypothesis is that loss of hippocampal neurons leads to remote deafferentation followed by gradual and progressive neuronal loss in connected regions. Impoverishment of hippocampal connections can lead to reduced complexity of remote circuitry [9]. The processes leading up to remote atrophy are also complex, and several mechanisms have been suggested [19]. Studies of the temporal and spatial sequencing of epilepsy and regional atrophy suggest that brain atrophy is a dynamic process that progresses over time [20]. In support of this hypothesis, it was noted that seizure spread and atrophy are unrelated [21], while white matter fiber integrity was correlated with remote atrophy [9]. Hence, the spread of both activity and atrophy are partially supported by prior studies, and a definite consensus eludes the field. The medial temporal lobe is highly connected and either case could theoretically lead to significant temporal atrophy. Since both modes of spread involve the anatomic connectivity network of the brain, a purely phenomenological or statistical analysis would not be able to disambiguate between them, as both would estimate similar topography, at least in the vicinity of the hippocampus. However, the brain-wide topography estimated by the two models are sufficiently distinct to allow quantitative disambiguation between the two, but this would require quantitative network modeling (see subsections Model I: Spread of Epileptogenic Activity and Model 2: Spread of Atrophy via Progressive Degenerative Process under Materials and Methods section).

The purpose of this paper is to develop network theoretic models of regional atrophy dynamics resulting from each of the above hypotheses, and to statistically determine which model is a better descriptor of the spatial patterning of real TLE atrophy. Both models are based on simple graph theoretic models of influence spread as a *Network Diffusion* (ND) process, enacted on the brain’s structural connectivity network whose nodes correspond to GM regions (obtained from atlas-based parcellation of T1-weighted MRI) and edges correspond to WM tracts between them (obtained from diffusion tensor imaging, DTI, followed by fiber tractography). The first model captures the network-centric spread of epileptogenic activity, using a recently developed network model of the spread of functional activity in brain networks [22]. Regional atrophy is treated as a simple consequence of established patterns of epileptic activity. This model only considers the time-averaged propensity of a region of experiencing epileptic activity, hence it does not preclude different patterns of seizure activity at different times in the same individual. The second model captures the spread of atrophy as a progressive degenerative process resulting from deafferentation. We show that this too can be adequately modeled by a very similar ND process, with the critical difference from the first model being that an initial atrophy seed is needed, putatively hippocampus in TLE-MTS, followed by a time-resolved progression of atrophy into distal regions. Epileptogenic activity does not enter this model, and deafferentiation is the primary propagating event. A similar model was employed to capture the spread of neurodegeneration in dementias [23]. Note that the complex neurobiology of excitotoxicty and remote degeneration cannot be tested using these macroscopic graph theoretic models. Our main contribution is to mathematically encode two competing but equally plausible mechanisms of spread, and show that they have differential macroscopic consequences which can be rigorously tested using imaging data alone, without requiring electrophysiology data. It is of course possible and likely that both excitotoxicity and deafferentation occur concomitantly in the same epileptic brain; this study is not designed to separate these two effects in an individual, but to judge, from group data, which of these two mechanisms plays a dominant role in the entire TLE population.

We tested the two models using group level atrophy distributions observed in two TLE cohorts: a) non-lesional temporal lobe epilepsy with mesial temporal sclerosis, and b) non-lesional temporal lobe epilepsy with no MRI-visible mesial temporal sclerosis. We show that while both models mimic group-level measured atrophy distribution, the (remote degeneration) spread-of-atrophy model performs better than the spread-of-activity model in both TLE-no and TLE-MTS groups. The study cohort, like the disease itself, contains considerable inter-subject variability, hence group-level tests will not be valid at the individual level. However, our purpose is not to apply this approach to individuals, but only to test which of two network models is better supported by group level data reflecting the relative involvement of different brain regions in the TLE population as a whole. Other potential etiologies of TLE atrophy unrelated to the network, including pre-existing conditions such as subtle cortical malformation microdysgenesis [24] or genetic variants that could be associated with specific atrophy patterns, are not considered in this study.

## Results

Computing the *t*-statistics between the TLE-MTS and TLE-no groups reveals no significant difference in atrophy, with some regions reaching significance threshold ±2std with the exception of the ipsilateral hippocampus, which is characteristic of TLE-MTS. Fig A in the S1 Text gives the *t*-statistics between the two groups and highlights the regions nearing significance. We fit the two proposed models, spread-of-activity (Model 1), and spread-of-atrophy via a progressive degenerative process (Model 2), to the two epilepsy subtypes (TLE-MTS and TLE-no). TLE subjects were grouped into ipsilateral and contralateral, by side-flipping one group, so that all patients had the epileptogenic focus on the left side, henceforth called the “ipsilateral side.” All named regions are understood to be from the ipsilateral side, unless otherwise specified. In all figures, the lobes are colored for ease of reading as follows: blue = frontal lobe, magenta = parietal lobe, red = occipital lobe, green = temporal lobe, cyan = cingulate structures, and black = subcortical structures. In this work, ventral diencephalon (VDC) refers to a group of structures that usually cannot be discriminated in standard T1-weighted images. The region includes the hypothalamus, mammillary body, subthalamic nuclei, substantia nigra, red nucleus, lateral geniculate nucleus, and medial geniculate nucleus. All eigen-modes **u**_*i*_ are ordered by the increasing associated eigenvalues such that **u**_1_ and **u**_86_ correspond respectively to the smallest and largest eigenvalues.

*Measured atrophy* is defined as the *t*-statistics obtained from the volumetrics of healthy group and each epileptic group, TLE-MTS and TLE-no, see Analysis Outline subsection for details. In this work model analysis is performed only at group level, individual subjects are not considered. In the case of Model 1 (spread of epileptic activity), both epilepsy types were bilaterally seeded in all temporal regions, followed by the evaluation of the model (Eq (10)) using a progressively increasing number of eigen-modes indexed by the corresponding eigenvalues ordered from smallest to largest, and the vector **x**_0_ has ones for the components corresponding to the temporal lobes of both hemispheres, and zero everywhere else. This model does not support systematic seeding of individual regions, since it is given in terms of brain-wide eigen-modes of the network. For each subset of the Laplacian eigen-modes the correlation *R* between the resulting atrophy estimate and the measured atrophy is computed. In Model 2 (atrophy via degenerative process) one node is seeded at a time, then as the diffusion into the network (*t* in Eq (14)) progresses, the correlation *R* between the empirical and the estimated atrophy is computed over a range of *t*. The estimated atrophy is then obtained from the *t* yielding the highest *R*. The process is thus repeated for all seed nodes. For example, the hippocampus is seeded, then the seed diffuses into the structural network as per Eq (14) as a function of *t*. The pattern yielding the highest *R* with the empirical atrophy is then used as the cortical atrophy estimate predicted atrophy. Thus, the results from Model 2 cover all regions, in turn, acting as seeds, and each such experiment is evaluated independently.

### TLE-MTS

**Fig 1:**
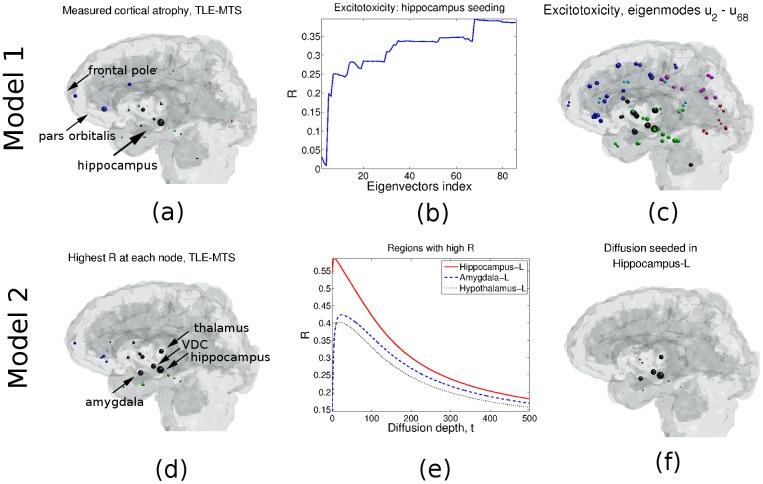
(a) TLE-MTS atrophy distribution. As expected, the hippocampus has the highest atrophy, consistent with TLE-MTS. (b) Pearson correlation *R* between Φ_1_ and measured atrophy vs. the number of eigen-modes used. Peak *R* is reached when eigen-modes **u**_2–68_ are used. (c) Atrophy distribution estimated using Model 1 using eigen-modes **u**_2_ − **u**_68_. Model 2: (d) Correlation *R* obtained when each node is seeded (model Φ_2_). The highest *R* is obtained when the hippocampus is seeded. (e) *R* vs. graph diffusion depth. Hippocampus seeding leads to the highest *R* is obtained at *t* = 5.56, followed by amygdala and the hypothalamus. (f) Estimated TLE-MTS atrophy obtained from Model 2 when the hippocampus is seeded.

The TLE-MTS measured atrophy reflected in Fig 1(a) indicates, as expected, pronounced atrophy in the hippocampus, with less marked atrophy ipsilaterally in pars orbitalis, amygdala, VDC, pallidum, thalamus and inferior temporal gyrus. Contralateral temporal pole, transverse temporal gyrus, entorhinal cortex (ERC) and pars opercularis also reveal some atrophy.

#### Model 1: Spread of epileptogenic activity

To evaluate this model (Φ_1_, Eq (10)), an initial seeding of epileptogenic activity is needed but unknown *a priori*. However, since the subjects are TLE we can safely assume that a temporal region is involved. To maintain generality and reduce bias, we will simultaneously seed all temporal regions in both hemispheres, and evaluate the summation in Eq (10) accordingly, for 2 ≤ *N* ≤ 86 terms successively. Fig 1(b) gives the Pearson correlation *R* of Φ_1_ and the empirical atrophy of TLE-MTS patients for each *N*. At *N* = 5 we observe the first, most substantial uptick in *R*. The best match is found at *N* = 68, giving *R* = 0.394 (*p* = 1.7 × 10^−4^). Using additional eigen-modes yields a small change downward in *R*, with *R* = 0.386 (*p* = 2.4 × 10^−4^) when *N* = 86 (Fig 1(b)). The largely monotonous increase of the *R* vs. *N* curve suggests that each eigen-mode makes an incremental but positive contribution to the accuracy of the model, but with diminishing returns coming from higher eigen-modes. Fig 1(c) gives the estimated atrophy distribution with *N* = 68 summation terms. The estimate indicates a strong peak at the hippocampus, followed by the thalamus and the amygdala. The model generally succeeds in capturing stereotyped MTS atrophy in ipsilateral regions, particularly in the temporal and frontal lobes. Most interestingly, it recapitulates the central and prominent role of the hippocampus in TLE-MTS based only on network eigen-modes.

**Fig 2:**
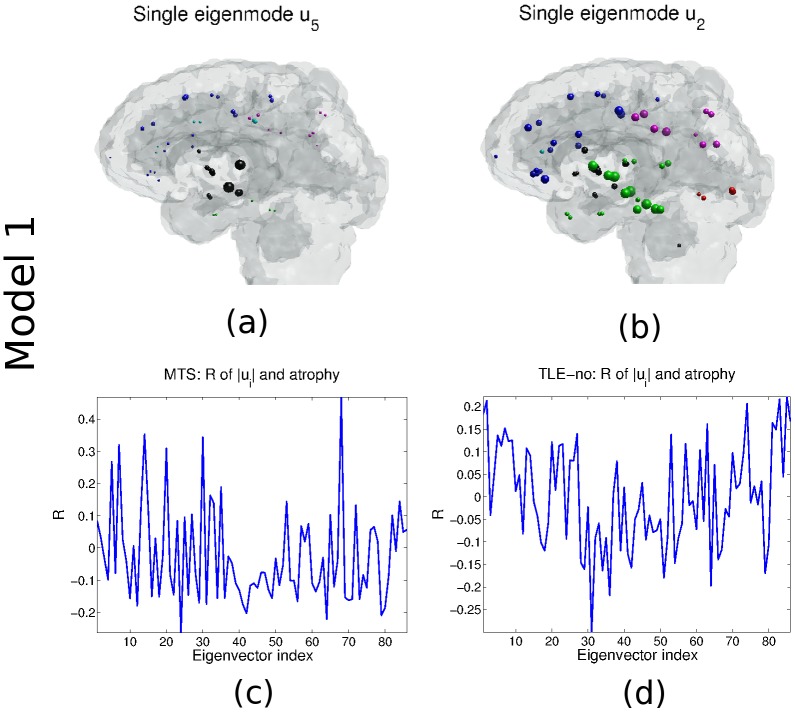
Model 1: Atrophy distribution via exitotoxicity. (a) Eigen-mode **u**_5_ captures the essentials of estimating network diffusion from the Laplacian’s eigen-modes for TLE-MTS when the ipsilateral hippocampus is seeded. (b) Eigen-mode **u**_2_ recovers features of the TLE-no when the temporal lobe is bilaterally seeded. (c) Plot of *R* vs the eigen-mode index for TLE-MTS when each eigen-mode **u**_*i*_ is correlated with the group atrophy. (d) Plot of *R* vs. the eigen-mode index for the TLE-no when eigen-modes **u**_*i*_ are each correlated with the group atrophy.

From Fig 1(b), the first eigen-mode to contribute meaningfully to the atrophy estimate is **u**_5_, with *R* = 0.01 (*p* = 0.94) when only eigen-modes **u**_2–4_ are used, and *R* = 0.198 when **u**_5_ (*p* = 0.07) is added. In fact, **u**_5_ shows a good similarity with measured *t*-statistics, with a bias for subcortical regions (Fig 2(a), with the largest coefficients of ∣**u**_5_∣ and corresponding regions listed in Table 1). In order to assess the contribution of individual eigen-modes to the model, Fig 2(c) gives the correlation *R* of the individual eigen-modes and the measured atrophy. Eigen-mode **u**_5_ yields *R* = 0.268 (*p* = 0.01), while **u**_68_ gives *R* = 0.469 (*p* = 5.4 × 10^−6^).

**Table 1:** Eigen-modes ∣**u**_5_∣ and ∣**u**_2_∣ (Fig 2(a) and 2(b)), and their dominant regions (Model 1).

Eigen-mode **u**_5_		Eigen-mode **u**_2_	
VDC-R	0.3170	Middletemporal-L	0.1844
VDC-L	0.3069	Superiortemporal-L	0.1803
Thalamus-R	0.2993	Inferiortemporal-L	0.1757
Thalamus-L	0.2804	Insula-L	0.1683
Hippocampus-L	0.2107	Fusiform-L	0.1682
Hippocampus-R	0.2058	Middletemporal-R	0.1671
Pallidum-R	0.1789	Insula-R	0.1660
Superiorfrontal-L	0.1639	Putamen-L	0.1655
Pallidum-L	0.1590	Superiortemporal-R	0.1673
Superiorfrontal-R	0.1584	Putamen-R	0.1625

#### Model 2: Spread of atrophy

Using Model 2, we estimate the distribution of network diffusion estimate Eq (14) and at each individual node compute its Pearson correlation *R* (see Materials and Methods section) against the TLE-MTS measured atrophy at all diffusion depths *t* with 900 evenly spaced samples in the interval [0, 100], and 100 evenly spaced samples in the interval [100.01, 500]. The process is repeated for all possible seedings, and the maximum *R* is obtained for each seeding—as shown in Fig 1(d). Ipsilateral hippocampus seeding appears to give the highest correlation, with *R* = 0.586 (*p* = 3.2 × 10^−9^), followed by the amygdala (*R* = 0.423, *p* = 4.9 × 10^−5^) and the hypothalamus (*R* = 0.402, *p* = 1.2 × 10^−4^). Other prominent candidate seed regions include thalamus, ERC and parahippocampal gyrus. In general the contralateral hemisphere gives small *R* for all of its nodes (in the range [0, 0.114]). The behavior of *R* versus diffusion depth *t* varies by seeding; some examples are shown in Fig 1(e) for three strongest candidate seeds: hippocampus, hypothalamus (part of VDC) and amygdala. The ND pattern obtained from hippocampal seeding of the model is shown in Fig 1(f).

Given that subcortical regions repeatedly show up as plausible seeds in our data and are widely conjectured as epileptogenic foci in related literature, we investigated the effect of subcortical seeding in more detail. We consider the network diffusions due to seeds located in the ipsilateral subcortical nodes for both epilepsy types, listed in Table 2. The cerebellum shows the weakest similarity *R*, suggesting secondary or insignificant involvement in seizure trigger or propagation in the epilepsy network. The TLE-MTS column gives the highest correlation at the hippocampus (*R* = 0.586), which is to be expected. High values for seedings at amygdala, thalamus and VDC indicates that these subcortical structures may have joint involvement in TLE-MTS etiology, supporting previous studies [25].

**Table 2:** Subcortical Pearson correlation *R* of the estimated atrophy and the *t*-statistics for both epilepsy types. “Max” refers to the overall highest *R* and the corresponding region, all located in the ipsilateral hemisphere.

	TLE-MTS	TLE-normal	Mean
Cerebellum	0.124	0.181	0.153
Thalamus	0.372	0.216	0.294
Caudate	0.225	0.229	0.227
Putamen	0.251	0.229	0.240
Pallidum	0.323	0.243	0.283
Hippocampus	0.586	0.210	0.398
Amygdala	0.423	0.194	0.309
Accumbens	0.232	0.227	0.230
VDC	0.402	0.213	0.308
Mean	0.326	0.216	
Max	0.586	0.243	
	Hippocampus	Pallidum	

### TLE-normal

**Fig 3:**
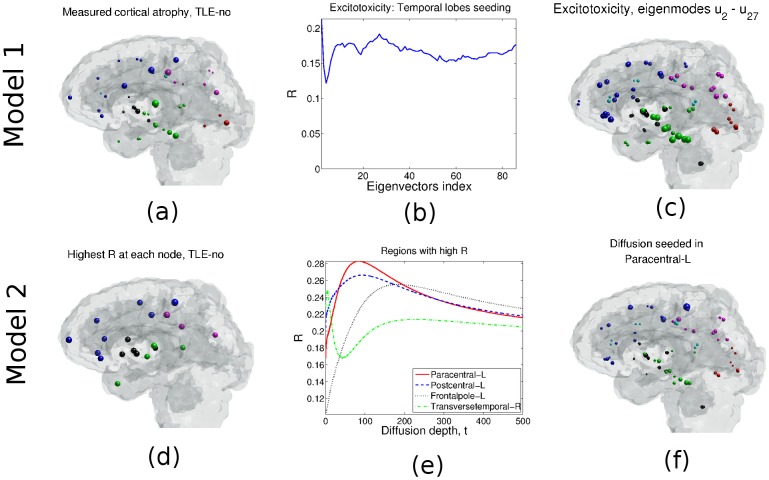
TLE-no case, Model 1: (a) Cortical/subcortical atrophy obtained from *t*-statistics of epileptic and healthy groups’ volumetrics. (b) *R* vs. the number of eigen-modes Eq (10) used for neuronal atrophy estimation. (c) Atrophy distribution estimated using Model 1 and eigen-modes **u**_2_ − **u**_27_. Model 2: (d) Correlation *R* of group atrophy and Φ_2_ obtained when each node is seeded. (e) *R* vs. graph diffusion depth *t*. Left paracentral gives the maximum *R*, followed by the left post central and the left frontal pole. (f) Neuronal atrophy estimate obtained from Model 2 when the paracentral lobe is seeded.

The measured atrophy of TLE-no, Fig 3(a), reveals pronounced atrophy broadly, bilaterally distributed across cortical and subcortical regions, particularly in the frontal and temporal lobes. The region with the highest atrophy is the contralateral transverse temporal gyrus, with *t*-statistics of 3.74. Other regions with high atrophy include the contralateral precentral gyrus (*t*-statistics 3.29) and the postcentral gyrus (*t*-statistics 3.45).

#### Model 1: Spread of epileptogenic activity

We estimate Φ_1_ from the network’s eigen-modes ${\left\{{\text{u}}_{i}\right\}}_{i=1}^{86}$ as described in Eq (10). The initial configuration comes from seeding all regions in the bilateral temporal lobes. Fig 3(b) gives the resulting Pearson correlation with the measured atrophy for the first *N* summation terms, 2 ≤ *N* ≤ 86. *R* reaches a maximum at eigen-mode **u**_2_, with *R* = 0.213 (*p* = 0.05), suggesting that eigen-mode **u**_2_ most closely approximates the atrophy distribution depicted in Fig 3(a). Using additional eigen-modes does not improve *R*, perhaps with the exception of eigen-mode **u**_27_, where we obtain *R* = 0.192 (*p* = 0.08). The resulting estimated atrophy pattern is depicted in Fig 3(c), implicating temporal lobe regions and the bilateral putamen. Due to the prominence of **u**_2_ in the above result, we investigated the pattern produced by **u**_2_ in Fig 2(b), with the largest coefficients of ∣**u**_2_∣ and corresponding regions listed in Table 1). The contribution of each individual eigen-mode to the TLE-no atrophy estimate is plotted against the eigen-modes’ index in Fig 2(d). As can be appreciated from the figure, **u**_2_ gives one of the highest Pearson correlations (*R* = 0.213, *p* = 0.05), along with eigen-modes **u**_83_ (*R* = 0.216, *p* = 0.05) and **u**_85_ (*R* = 0.222, *p* = 0.04) with comparable *R* values.

#### Model 2: Spread of atrophy

Pearson correlation analysis of the measured atrophy and the estimated atrophy obtained from Model 2 Eq (14) when seeded at each node is performed. Fig 3(d) depicts the distribution of maximum *R* after seeding each region in turn, and suggests that in TLE-normal group, there is no strong evidence of prominent and stereotyped seeding. While the obtained coefficients *R* for all seeds are comparable, a seed in the paracentral sulcus (frontal lobe) gives the highest correlation (*R* = 0.283, *p* = 8.3 × 10^−4^). The postcentral gyrus gives the second highest correlation, with *R* = 0.266 (*p* = 0.013). Fig 3(e) gives the curve of *R* vs. diffusion time *t* when a seed is placed in the postcentral gyrus. The maximum *R* = 0.283 is reached at *t* = 72.53. For comparison we also show *R* curves corresponding to other plausible seedings: hippocampus, which, unlike MTS case, does not exhibit a pre-eminent role as seed region; and contralateral transverse-temporal gyrus, which is probably an empirical aberration, a view cemented by the anomalous *R* curve it exhibits. The estimated atrophy from the model seeded at paracentral gyrus, evaluated at maximum *R*, is shown in Fig 3(f), and implicates subcortical, temporal, frontal and parietal regions, in that order. Bilateral fusiform and middle temporal gyri, inferior and superior temporal gyri, and the precentral gyrus stand out. We note that the spread-of-atrophy model underestimates the atrophy in the occipital lobe and misses the contralateral pars opercularis and precentral gyri in the frontal lobe. Whilst the contralateral transverse temporal gyrus suffers a significant atrophy (Fig 3(a)), it appears to be largely missed in the ND model (Fig 3(f)).

Referring to Table 2, we notice that for the TLE-no case the ipsilateral subcortical regions caudate, putamen, pallidum, and the accumbens *R* coefficients are quite comparable with the maximum *R* obtained in the paracentral gyrus. From this result it is difficult to assign a dominant or focal onset zone. This underscores that the TLE-no group is not only heterogeneous in its atrophy topography, but also in the site of epilepsy origin. This means that group level fitting presented here has limited utility for the heterogeneous TLE-no case compared to TLE-MTS, where the hippocampus was revealed as the strongest likely region of origin.

### Models’ Accuracy

**Fig 4:**
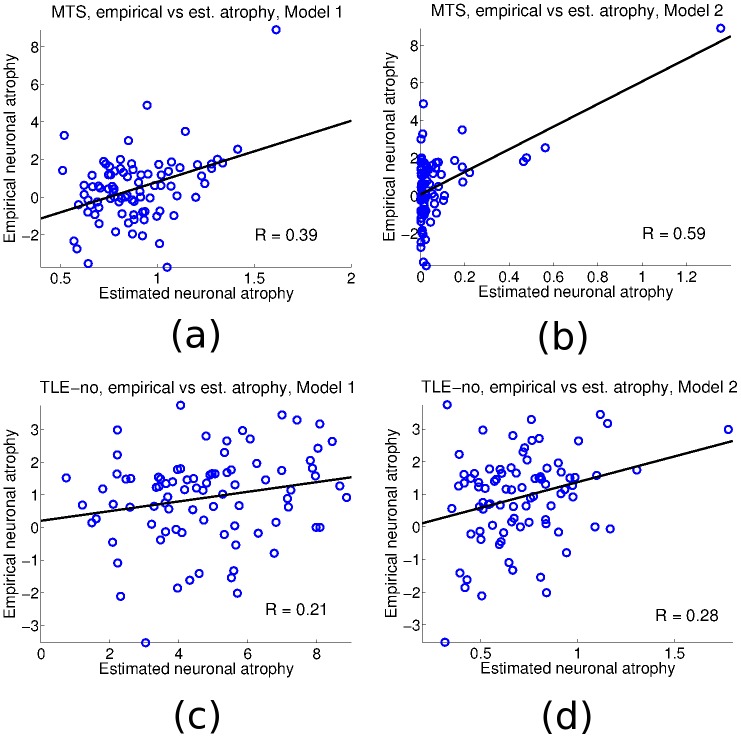
Scatter plots for each model and epilepsy type of the measured neuronal atrophy vs. the neuronal atrophy as estimated by the two models. Empirical vs. estimated neuronal atrophy for the case of TLE-MTS; Model 1 (a), and Model 2 (b); and measured vs. estimated neuronal atrophy for the case of TLE-no; Model 1 (c), and Model 2 (d). For both types of epilepsy, Model 2 outperforms Model 1.

To visualize the possibility of outliers or anomalous distribution of points in both models, we scatter plot the measured neuronal atrophy vs. its estimate for both models and both types of epilepsy. Fig 4(a) and 4(b) give the scatter plots for the case of TLE-MTS Models 1 and 2 respectively. The Model 1 plot reveals more widely scattered points than in the case of Model 2, where the model is more selective in its estimate of atrophy. This is also consistent with the higher *R* obtained from Model 2. Similar observations can be made for the TLE-no case, Fig 4(c) and 4(d); while the plots are more scattered than in the TLE-MTS case above, Model 2 still gives a more accurate estimate of the neuronal atrophy than Model 1. In order to further explore the models’ sensitivity to outliers, Fig B in S1 Text gives the scatter plots of the empirical atrophy vs the logarithm of the estimated atrophy. The resulting *R* is computed for all four cases. As reflected in the figure, Model 2 is once again superior to Model 1 for both TLE-MTS (*R* = 0.400 vs *R* = 0.340, Figs. B(a) and B(b)), and TLE-no (*R* = 0.270 vs *R* = 0.170, Figs. B(c) and B(d)).

### Null Model

To rule out the possibility that the above results were obtained by chance, we predict a randomly shuffled version of the measured atrophy for both types of epilepsy using both Models 1 and 2. For each model and each epilepsy type we run 1,000 iterations of randomly shuffled versions of the measured atrophy and compute the resulting *R*. For Model 1, in each iteration we find *R* for Φ_1_ Eq (10) with the sum evaluated up to *K* with 2 ≤ *K* ≤ 86. For Model 2, we evaluate Φ_2_ Eq (14) over a range of *t* when the hippocampus is seeded (case TLE-MTS), and when the paracentral sulcus is seeded (case TLE-no). For each iteration, the correlation *R* is computed in a similar fashion to sections TLE-MTS and TLE-normal above.

**Fig 5:**
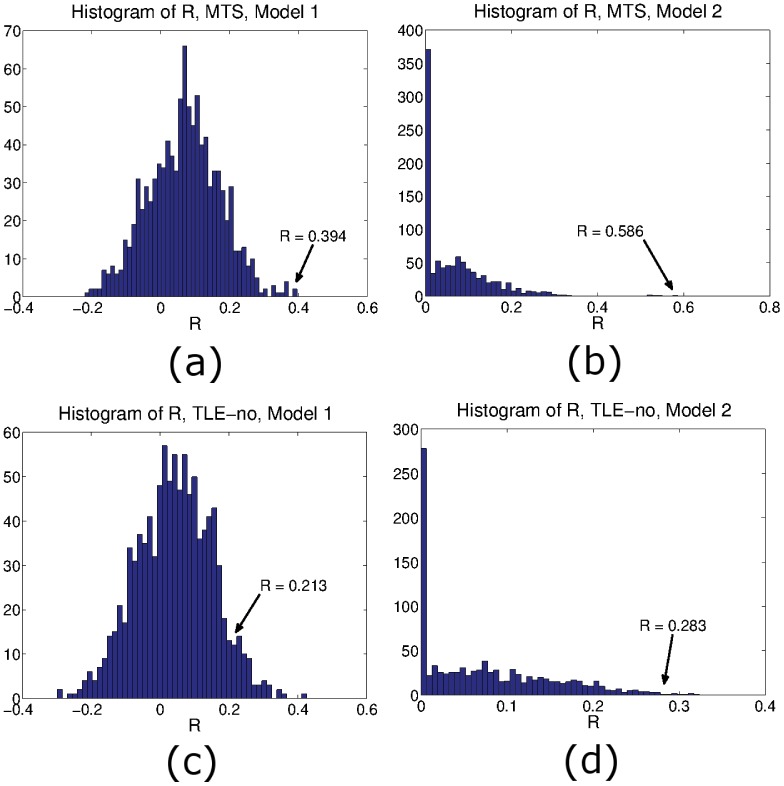
Histograms of *R* resulting from 1,000 instances of random permutations of the neuronal atrophy for each type of epilepsy and for both models. From the histograms, the estimated neuronal atrophy is likely to be specific to the atrophies obtained from both epilepsy groups, more so in the case of Model 2 where high *R* is obtained for both types of epilepsy. Histograms of *R* resulting from random permutation of neuronal atrophy, (a) TLE-MTS Model 1, (b) TLE-MTS, Model 2. Histogram of *R* resulting from TLE-no neuronal atrophy random permutation, (c) Model 1, and (d) Model 2.

Fig 5 summarizes the findings. Fig 5(a) indicates the histogram resulting from the randomly shuffled measured atrophy in the case of Model 1 and TLE-MTS. None of the shuffled atrophies achieved a correlation exceeding that obtained from the empirical measured atrophy (*R* = 0.394). Similarly, measured atrophy randomization in Model 2 gives a histogram where the measured atrophy has the highest *R*, Fig 5(b). The TLE-no case does not perform as well. Fig 5(c) gives the histogram obtained in the case of Model 1. The measured atrophy has a correlation of *R* = 0.213, with 57 randomly shuffled instances of the atrophy yielding higher *R*, with a maximum *R* = 0.426. Nonetheless, only a small percentage of all iterations, 5.7% give *R* > 0.213. Model 2 gives improved results, with only 8 random atrophy patterns having *R* > 0.283 (0.8% of all iterations). The highest correlation obtained is *R* = 0.323. The fact that all reported *R* values are at the extreme end of the null distribution conveys a strong suggestion that the network is indeed a relevant modulator or atrophy in TLE. The fact that the reported *R* statistic of Model 1, but not of Model 2, is only moderate in relation to the null histogram further cements our conclusion that Model 2 (degenerative spread) is a superior network model than Model 1 (excitotoxicity spread).

## Discussion

Understanding where an epileptic seizure originates, which regions undergo ictal and interictal hyperactivity, how and where in the brain seizure activity causes atrophy are important questions in epilepsy research. Both effective models of seizure propagation as well as a thorough understanding of how epilepsy induces cortical atrophy are lacking as of yet. A close correspondence exists between the sites of pronounced hyperactivity and atrophy patterns [4, 26], but two competing hypotheses can explain these findings: 1) hyperactivity propagates in connected brain regions and via local excitotoxicity causes atrophy [11, 13–15]; 2) excitotoxicity in the onset zone causes atrophy, which then propagates to connected regions via a deafferentation and remote degeneration process involving a cascade of neurobiological events leading to frank neuronal death [9, 19–21]. Existing neuroimaging studies are mixed [1–6, 9, 11, 13–15, 20], some finding support for one model, and some for the other. But quantitative model-based testing of these competing views has not been reported previously.

In this paper we attempt such a study, by mathematically encoding each competing hypothesis in terms of network spread, and testing them on measured atrophy data in TLE. The first model captures the spread of epileptogenic hyperactivity as a network diffusion process, based on the following assumptions: a) epileptogenic activity in epileptogenic regions induces a similar activity in distal but connected regions, b) this influence is stronger between highly connected regions, c) once a region begins to experience epileptogenic activity, it can then act as a seed for propagating this process to other regions, and d) atrophy is the excitotoxicity-driven consequence of lifetime hyperactivity. EEG data lend strong support to this model. In TLE, partial loss of consciousness, known as complex partial seizures, is induced by the hyperactivity starting in temporal regions and spreading to wider brain networks, well past the ictal period. While each patient and even seizures in the same patient can be different, recordings during and after ictal activity frequently show progression of activity from medial to lateral temporal, followed by orbitofrontal and frontoparietal regions [27]. In the extreme case, when ictal activity turns into a generalized seizure and reaches the contralateral temporal lobe, this can lead to loss of consciousness [28]. While this process is complex and locally nonlinear, we expect that its overall behavior can be *grosso modo* approximated by linear network-based dynamic models driven by low-order differential equations. Such models are already popular in areas of signal and image processing [29, 30], and have recently been applied to model the spread of functional activity in brain networks [22]. The second model assumes that atrophy is caused by progressive deafferentiation, which is the primary propagating event, recapitulating classic neurodegenerative progression along fiber tracts, via a complex neurobiological cascade, which on a macroscopic scale is again simplified into a linear dynamic process.

Both quantitative network diffusion models developed here yield similar first order differential equations constrained on the network, giving similar network dynamics, and each model has only one degree of freedom, *β* or *γ*. Although this succeeds in reducing the complex spatio-temporal dynamics of epileptogenic atrophy of each spread hypothesis to a highly parsimonious, low-dimensional model, it also underscores the difficulty in disambiguating between them. Both models give a strong resemblance to measured group level atrophy data from two TLE cohorts—29 TLE-MTS subjects and 50 TLE-normal subjects. Due to its highly localized atrophy (ipsilateral hippocampus), TLE-MTS yields the best atrophy estimation with high *R*, when seeded at the hippocampus (Fig 1(d)–1(f)). However, both subtypes TLE-MTS and TLE-no show validation of both models, whose estimates are significantly correlated with measured atrophy and appear to be consistent with similar conclusions reached in the literature. On the whole, however, our data favors the degenerative spread-of-atrophy model over the spread-of-activity model; in TLE-MTS: *R* = 0.586 (hippocampal seeding, Model 2) versus *R* = 0.396 (**u**_2–68_, Model 1); in TLE-no: *R* = 0.283 (paracentral gyrus seeding, Model 2) versus *R* = 0.213 (**u**_2_, Model 1). As described earlier, a simple test of which model is better is whether the fit with real data peaks at an intermediate value of *t* or whether the fit is monotonically increasing in *t*, peaking at *t* = ∞. The behavior of the *R* vs. *t* curves in Figs 1(e) and 3(e), is clearly not monotonic, instead peaking at intermediate values of *t*. This supports the above conclusion, that spread-of-atrophy (Model 2) is more plausible than Model 1. We note that the prominence of paracentral gyrus as seed in TLE-no is surprising; while epileptic seizures originating in this region are common and usually accompanied by motor symptoms, they are not typically classified as TLE. Hence our result is most likely due to the extremely strong precentral atrophy seen in some of our TLE-no cohort, and/or the strong connectivity between precentral and various subcortical structures. Indeed, subcortical regions were found to act almost equally well as seed regions in our models (Table 2), which is much more plausible. The models’ simplicity leads to a fast implementation with minimal computational power. This study is meant as a proof of concept rather than as a clinical application, hence its use of group atrophy rather than individual subject data. However, successful testing of the presented network dynamic models could open the door to a number of potential clinical applications on *individual patients*, including estimation of future atrophy, identification of seizure onset zones and surgical planning.

### Small Eigen-modes as Attractors and Modulators of Epilepsy

The brain graph’s Laplacians eigen-modes should be intuitively understood as sub-networks within which an excitotoxicity-driven process would get “trapped” and reverberate. Network eigen-modes are increasingly appearing in brain science; for instance, they act as attractors for dementia pathology [23]. A description of the neuroscientific meaning of these eigen-modes, as applied to brain activity spread, was given in our previous paper on this topic—see [22].

Our results support a somewhat speculative role of network eigen-modes in epileptic processes, although we note that this study was not designed to explore this aspect in detail. Our theory finds that atrophy (and activity) would be predominantly contained within a few small spatially distributed but separate sub-networks of the brain, given by the eigen-modes of the network Laplacian–Eqs (10) and (14). Closer inspection of selected small eigen-modes (Fig 2(a) and 2(b)) reveals a compelling picture whereby **u**_2_ matches the spatial pattern of TLE-no atrophy, and **u**_5_ matches TLE-MTS. Both eigen-modes are dominated by temporal, medial and subcortical structures—precisely the regions that are most frequently associated with TLE. To be sure, other eigen-modes also bear resemblance to atrophy data (Fig 2(c) and 2(d)), but due to their large eigen-value they do not contribute significantly to either model.

The marked predilection of epilepsies to occur in medial temporal and subcortical regions is well known, but poorly understood more than 60% of all epilepsies are of the temporal type, and of those 80% have MTS (statistics from epilepsy.com). Why are certain regions especially vulnerable to epileptic seizures, and why are the patterns of atrophy in certain epilepsies so stereotyped? Given that seizures can in theory arise in any part of the brain (as they frequently do, in frontal, parietal and other regions), that seizures frequently generalize outside of onset zones, and sometimes even recruit the entire brain, the principle of parsimony would dictate that epilepsies should recruit all regions equally. That this is not so suggests that vulnerable structures enjoy a privileged location in the brain network. However, these regions do not stand out in terms of degree, clustering, path length or other conventional network theory metrics. That small network eigen-modes consistently implicate these regions, which are central actors in epileptogenic activity and atrophy, could provide a potential explanation for regional and stereotyped predilection of TLE, and why it happens to be the most common variant of focal epilepsy [25]. Thus, temporal, limbic and subcortical regions are located in a network neighborhood that anchors some of the smallest network eigen-modes, which act as attractors for epileptogenic activity or degenerative processes. This interpretation of small eigen-modes was first put forward in [23] in the context of neurodegenerative disease modeling, and current results suggest a similar, albeit speculative role in epilepsy.

### Agreement with Prior Studies in TLE Atrophy

Similar to previous morphometric results [5, 31, 32], the spread-of-activity model (Model 1) finds widespread atrophy in the ipsilateral temporal lobe (fusiform and parahippocampal). In addition to the regions outlined in [5] for TLE-MTS, Model 1 finds atrophy in the ipsilateral middle temporal gyrus, lateral orbital frontal gyrus, and precentral and postcentral gyri. The model also finds significant subcortical atrophy, particularly in the hypothalamus, amygdala, and hippocampus. High atrophy is also found in the contralateral thalamus and hippocampus. TLE-MTS atrophy is also widely reported in the frontal lobe [33] where it is proposed that under certain conditions mesiotemporal epileptic activities would propagate through the thalamus to the frontocentral areas. Frontal and parietal regions play a crucial role in the evolution of complex partial seizure [28], and Model 1 (but not Model 2) captures this, especially in lateral orbitofrontal regions. On the other hand Model 2 reveals atrophy patterns chiefly in the subcortical ipsilateral region, chiefly the hippocampus, amygdala, hypothalamus, and thalamus, all strongly implicated in epileptogenesis [5, 32]. An additional region captured by Model 2 (but not Model 1), is ERC (Fig 1(f)).

TLE-normal atrophy is known to be widespread bilaterally, with no apparent focus nor sclerosis visible on MRI [5, 34, 35]. Both network models give bilateral effects without a clear focus. In this group, the progressive degeneration model is not as strongly supported as for the MTS group, since the highest *R* from seeding various regions does not show a clear winner (Fig 3(d)). The most plausible seeds are precentral gyrus and frontal pole—neither being an especially prominent source of either seizure activity or atrophy in TLE. The progressive degenerative model with precentral seeding largely agrees with previously published results [5, 34, 35], with significant atrophy found in the ipsilateral inferior temporal gyrus, an epileptogenic focus candidate [5]. Regions with significant estimated atrophy not reported in [5] include the contralateral fusiform and the ipsilateral superior frontal gyrus. The latter node is structurally connected with the ipsilateral putamen where the proposed network diffusion model estimates high atrophy.

### Relationship to Prior Work in Brain Network Analysis

This study is related to but distinct from conventional analysis of epilepsy networks [6, 17, 36–39] or functional connectivity analyses [40–42]. Additionally, the effect of TLE on the structural network topology has been well studied, see for example [43–46]. Here our focus is in developing models of networked spread from first principles, rather than obtaining descriptive network statistics for epilepsy networks. The general class of network dynamics developed here, *network diffusion*, is well known in the area of signal and image processing [29, 30]. For example it has been used for image enhancement [29] using weighted graphs. In [30] the authors propose an image smoothing method using heat kernel and weighted undirected network. Bougleux *et al* consider discrete image smoothing and denoising on weighted graphs using Laplacian operators [47]. Our laboratory was the first to develop diffusive dynamics enacted on networks as a macroscopic model of the evolution of neurodegenerative brain diseases like Alzheimer’s disease and other dementias [23]. However this does not imply any similarity in pathophysiological mechanisms between dementia and epilepsy, two different disease processes. The spread-of-activity model is based on our prior work on modeling the patterns of functional connectivity in the brain [22], where we hypothesized that on a given structural network, functional influences must travel along connected edges, and satisfy first-order diffusive dynamics which are natural for this kind of influence propagation. The ability of a common mechanistic network model to mimic disparate brain behaviors, from neurodegenerative spread to functional correlations to epilepsy does suggest a striking convergence in the behavior of large scale brain phenomena.

### Study Limitations and Future Work

Conceptually, the prime limitation of this study is that it assumes no cross-talk between the two alternative models of spread, activity and atrophy—necessitated by the purpose of testing which model “wins”. It is not known whether this is strictly accurate, and several reports suggest evidence of interplay [9, 19–21].

However, we believe that a statistically sound first step mandates that the two mechanisms be tested and judged separately before potential interplay can be realistically modeled. Additional effects such as developmental abnormalities may come into play. This however is always an issue in any disease with a wide spectrum of causative factors, such as epilepsy. This should not, and does not, reduce the utility of network modeling efforts as long as one can strongly believe that the network is modulating the advance of disease, regardless of etiology. There is no doubt that such is the case with epilepsy. We emphasize that the presented models cannot address disease etiology or pathophysiology; its value lies in showing that the macroscopic effect of network dynamics can largely explain the stereotyped patterns of atrophy in TLE, regardless of individual subjects etiologic factors.

Another limitation is that while we model the spread of activity, we do not utilize electrophysiological data to test this, instead relying on regional atrophy patterns. This too is necessitated by the current goal of testing between competing models, but in the future it would be desirable to explore the spatiotemporal dynamics of EEG recordings. For this work the whole brain EEG data needed to achieve network modeling has proven exceedingly difficult to acquire on our patients. Current connectome technology based on diffusion MRI and tractography underestimates long-range connections, and is especially unreliable at estimating inter-hemispheric connections and tracts passing through complicated neighborhoods, for instance frontotemporal connections via the uncinate fasciculus and the connections between the limbic system and orbitofrontal cortex. As a result the model’s estimated atrophy shows bias for ipsilateral hemisphere and underestimates contralateral atrophy. Orbitofrontal cortex is strongly connected to limbic structures and shows significant atrophy in both TLE-MTS and TLE-no cases, but is underestimated by our models due to underweighted uncinate fasciculus connectivity. Another limitation is that our models assume static connections and do not account for change in connections due to alteration in activity or atrophy. Since this study involves measured atrophy, we could only include epilepsy subtypes TLE-MTS and TLE-no which have stereotyped topography of atrophy. A preliminary investigation of cases of more heterogeneous epilepsies gave inconsistent patterns amongst subjects as expected, and made the model correspondingly non-informative. Hence proposed models may not be applied at group level to heterogeneous epilepsies.

## Materials and Methods

### Ethics Statement

This study was approved by UCSF’s IRB and written informed consent was obtained from each subject according to the Declaration of Helsinki. Seventy nine patients suffering from drug resistant TLE were recruited between mid 2005 and end of 2007 from the Pacific Epilepsy Program, California Pacific Medical Center and the Northern California Comprehensive Epilepsy Center, UCSF, where they underwent evaluation for epilepsy surgery.

### Network Notation

In a brain network each node represents a GM region located on either the neocortex or in deep brain subcortical areas. We define a network 𝒢=(𝒱,ℰ) with a set of *N* nodes given by $𝒱=\{v_{i}|i\in 1,\ldots ,N\}$ and a set of edges given by an ordered node pair ℰ={(i,j)∣i∈𝒱,j∈𝒱} [48]. Between any two nodes *i* and *j* there might exist an edge representing a fiber tract whose connectivity weight *c*_*i*,*j*_ ∈ [0, ∞) can be measured from the streamlines of the dMRI tractography, and this defines a connectivity matrix $\text{C}=\{c_{i,j}|(i,j)\in ℰ\}$. Although some individual neurons are known to be directional, dMRI does not allow measurement of directionality. Major fiber bundles resolvable by dMRI, especially cortico-cortical pathways are generally bidirectional, having roughly equal number of connections in either direction [49]. We define the *connectivity strength* or the *weighted degree* of a node *i* in this network as the sum of all connection weights:

$$\begin{array}{c} \delta_{i}=\sum_{j|(i,j)\in E}c_{i,j}. \end{array}$$ (1)

Table 3 describes the various parameters and variables used in this work.

**Table 3:** Summary of the variables and definitions used in this text.

Parameter or variable	Role
𝒢	structural network of *N* nodes
𝒱	set of nodes of 𝒢
*v*_*i*_	*i*th node of 𝒢
ℰ	set of edges of 𝒢
**C**	structural connectivity matrix
*c*_*i*,*j*_	element (*i*, *j*) of **C**
*δ*_*i*_	weighted degree of node *i*
ℒ	Laplacian of **C**
*λ*_*i*_	*i*th eigenvalue of ℒ
**u**_*i*_	*i*th eigen-mode of ℒ
Δ	diagonal degree matrix
*β*	Model 1 diffusion rate
*γ*	Model 2 diffusion rate
*x*_*i*_(*t*)	hyperactive neurons in a given volume *i*
*V*_*i*_	number of voxels in *i*th region *Ri*
Φ_1_	atrophy spread due to excitotoxicity
*y*_*i*_(*t*)	neuronal loss in the *i* region
Φ_2_	atrophy spread due to neuronal loss

### Model 1: Spread of Epileptogenic Activity

We codify here, from first principles, the hypothesis that epileptogenic activity in the epileptogenic focus spreads outward following known anatomical pathways. What is described here is a simple diffusion process, a model that applies to any spreading quantity. Here, that quantity is brain activity, and we model how it would propagate along the structural network of the brain. For an isolated GM region *R*1, the average activation signal over all its neurons *x*_1_(*t*) is proportional to the number of hyperactive neurons per voxel. We assume a simple damped system behavior, given by d*x*_1_(*t*)/d*t* = −*βx*_1_(*t*). This damped behavior is consistent with epileptogenic activity, which has a transient nature and dies away exponentially. Damping in such a system may be assumed to arise from gradual loss of synchrony due to dephasing between neurons. Damping behavior of this sort has a long history in brain signal modeling, especially in neural mass approximations of neuronal assemblies [50], firing rate neural models [51], and is ultimately governed by membrane time constant of leaky-integrate-and-fire neurons [52] in all these exponential signal decay is the key component. Next consider a *pair* of cortical regions *R*1 and *R*2, whose connectivity weight is *c*_1,2_. The number of hyperactive neurons in *R*2 is proportional *V*_2_ *x*_2_, where *V*_2_ is the number of voxels in *R*2. Of these, the number of axonal projections from *R*2 to *R*1 is proportional to $c_{1,2}\frac{1}{\delta_{2}}V_{2}x_{2}$, where we divide the connectivity by the weight of *R*2, *δ*_2_ (see Eq (1), to get a ratio. The proportion of neurons in *R*1 which experience a hyperactive afferent from *R*2, assuming uniform mixing of afferents, is then given by $\frac{1}{V_{1}}c_{1,2}\frac{1}{\delta_{2}}V_{2}x_{2}$. Our key assumption next is that each afferent hyperactive neuron is able to induce hyperactivity in connecting neurons at a constant rate, modeled as a Poisson process. Therefore, the rate of change in the number of *R*1 neurons which undergo hyperactivation secondary to enervation by *R*2, after accounting for the internal (damped) dynamics of *R*1, is given by

$$\begin{array}{c} \frac{dx_{1}\left(t\right)}{dt}=\beta (\frac{1}{V_{1}}c_{1,2}\frac{1}{\delta_{2}}V_{2}x_{2}\left(t\right)-x_{1}\left(t\right)) \end{array}$$ (2)

where we assume identical rate constant *β* for both the internal and external signals contributing to the dynamics of *R*1 for simplicity. For multiple afferents into *R*1, we modify this to

$$\begin{array}{c} \frac{dx_{i}\left(t\right)}{dt}=\beta (\frac{1}{V_{i}}\sum_{j}c_{i,j}\frac{1}{\delta_{j}}V_{j}x_{j}\left(t\right)-x_{i}\left(t\right)). \end{array}$$ (3)

Assuming a previously proposed relationship between GM volume and degree given by $V_{k}\propto \sqrt{\delta_{k}}\;\forall k\in 𝒱$ [22] we have

$$\begin{array}{c} \frac{dx_{i}\left(t\right)}{dt}=\beta (\delta_{i}^{-\frac{1}{2}}\sum_{j}c_{i,j}\delta_{j}^{-\frac{1}{2}}x_{j}\left(t\right)-x_{i}\left(t\right)). \end{array}$$ (4)

Expanding to include the entire network, Eq (4) can be expressed compactly as:

$$\begin{array}{c} \frac{dx(t)}{dt}=-\beta Lx\left(t\right), \end{array}$$ (5)

where **x**(*t*) is an *N* × 1 vector describes the fraction of hyperactive epileptogenic neurons in all brain regions, and the matrix ℒ is the well-known symmetric and nonnegative definite network Laplacian

$$\begin{array}{c} L=I-\Delta^{-1/2}C\Delta^{-1/2}, \end{array}$$ (6)

where Δ is the diagonal degree matrix with the node degree *δ*_*i*_ as the *i*th diagonal element.

A closed form solution to Eq (5) is given by

$$\begin{array}{c} x\left(t\right)=\exp\left(-\beta Lt\right)x_{0}. \end{array}$$ (7)

Details of relevant network theory can be found in [48]. This describes the first order dynamics of the spread of epileptogenic neuronal populations along brain networks, starting at an initial configuration **x**_0_. The ND model above is *mass conserving*, entailing no increase of hyperactivity overall, merely its distribution from focal loci to wider networks. This is appropriate for TLE, where ictal activity in medial temporal regions rarely generalizes systemically or leads to a total loss of consciousness [28]. It is important to note that the time-scale of above equations is related to neural signal transmission, measured in milliseconds.

The spread-of-activity model assumes that atrophy in any brain region is the consequence of excitotoxicity induced by lifetime epileptogenic activity in that region. The process of neuronal death secondary to hyperactivity is not fully understood, and several processes have been implicated, including trophic exchanges, remodeling of brain networks [17], and complex cascade of neurobiological events [18]. At a macroscopic level, however, these complexities may not be germane, and the overall accumulative effect on atrophy may simply be captured by:

$$\begin{array}{c} \Phi_{1}\propto \int_{0}^{\infty }x\left(\tau \right)d\tau . \end{array}$$ (8)

In this model, the stronger and more frequent the level of hyperactivity experienced by a region, the more atrophy it will suffer. Although a full verification of the network diffusion model of activity spread would require multi-channel ictal and interictal EEG measurements, using the above model relating activity to atrophy we are able to circumvent this, and instead utilize MRI-derived regional atrophy as the end measure of the model.

Due to integration to ∞, atrophy in this model does not explicitly depend on diffusion depth *t*. Expanding Eq (8) in terms of Laplacian eigen-modes and exchanging the order of summation and integration:

$$\begin{array}{c} \Phi_{1}=\sum_{i=1}^{N}u_{i}u_{i}^{\prime }x_{0}\int_{0}^{\infty }e^{-\beta \lambda_{i}t}dt, \end{array}$$ (9)

and where {*λ*_*i*_} are the eigenvalues of the Laplacian matrix ℒ, and {**u**_*i*_} are the corresponding eigen-modes. Evaluating the definite integral above we obtain

$$\begin{array}{c} \Phi_{1}=\frac{1}{\beta }\sum_{i=1}^{N}\frac{1}{\lambda_{i}}u_{i}u_{i}^{\prime }x_{0}=\frac{1}{\beta }L^{†}x_{0}, \end{array}$$ (10)

where we consider only the terms with *i* > 1, since the first eigen-mode simply represents uniform activation of the entire brain, which is not relevant in TLE, which rarely displays generalized epileptogenic activity that recruits the entire brain. ${ℒ}^{†}$ refers to the interesting observation that the above expression evaluates to the pseudo-inverse of ℒ. Further, due to the $\frac{1}{\lambda_{i}}$ term, the smallest eigenvalues have the most dominant contribution, thus the summation above is only needed for the first few eigen-modes. In Results section, we evaluate the similarity between measured atrophy patterns and Φ_1_ over varying number of eigen-components.

### Model 2: Spread of Atrophy via Progressive Degenerative Process

This model assumes that rather than epileptic activity the process of progressive deafferentation is the propagating event, a process initiated at onset zones by excitotoxicity and other causes, but which thereafter spreads throughout the brain network via remote degeneration. Again, the complex neurobiological cascades leading to frank remote degeneration, involving axonal reaction, inflammation, autophagy, oxidative damage, synaptic dysfunction, loss of trophic support, anterograde and retrograde degeneration, and finally frank neuronal death [19] are not attempted to be modeled here. Instead, we assume that on a macroscopic level, these details are not germane, and that the overall behavior is governed by linear dynamics. Given that degeneration is a result of slow processes which build up over time rather than act instantaneously, it is appropriate to model the local (to the region) degenerative dynamics set in motion by a single event entailing loss of $y_{1}^{0}$ neurons per voxel in an isolated region *R*1 via an impulse response function, which is commonly used in linear systems theory to characterize such non-instantaneous effects [53]. Since the exact shape of this impulse response is not known, we choose one of the simplest but most widely used plausible causal functions, given by the exponential decay function $y_{1}(t)=y_{1}^{0}\text{exp}(-\gamma t)$, which simply encodes the expectation that a single insult will cause further but non-instantaneous degeneration, persisting in the region with a half-life of 1/*γ*. This corresponds again to a simple damped system behavior, which may be written as d*y*_1_(*t*)/d*t* = −*γy*_1_(*t*). Next consider a *pair* of cortical regions *R*1 and *R*2, whose connectivity weight is *c*_1,2_. In a short time interval *δt*, let the number of newly deceased neurons in *R*2 be *V*_2_ *y*_2_, where *V*_2_ is the number of voxels in *R*2. This group of external newly deceased neurons then cause degeneration in *R*1, modeled as a Poisson process in close analogy to Model 1. Thus, accounting for both the internal and externally-induced atrophy dynamics in *R*1, we have

$$\begin{array}{c} \frac{dy_{1}\left(t\right)}{dt}=\gamma (\frac{1}{V_{1}}c_{1,2}\frac{1}{\delta_{2}}V_{2}y_{2}\left(t\right)-y_{1}\left(t\right)) \end{array}$$ (11)

where we assume identical rate constant *γ* for both the internal and external atrophy dynamics for simplicity. Since these equations are fully analogous to Model 1, on the entire network we have

$$\begin{array}{c} \frac{dy(t)}{dt}=-\gamma Ly\left(t\right), \end{array}$$ (12)

where **y**(*t*) is an *N* × 1 vector describes the density per voxel of dying neurons in all brain regions. Eq (12) admits a closed form solution $\text{y}(t)=\text{exp}(-\gamma ℒt){\text{y}}_{0}$, giving a time-dependent process starting with initial “seed” map **y**_**0**_ at *t* = 0, and ending at a uniform distribution at *t* = ∞. Since **y**(*t*) denotes the number of newly deceased neurons at any instant, the overall atrophy during the degenerative process is given by the time integral

$$\begin{array}{c} \Phi_{2}\left(t\right)\propto \int_{0}^{t}y\left(\tau \right)d\tau , \end{array}$$ (13)

which has a closed form solution

$$\begin{array}{c} \Phi_{2}\left(t\right)=\frac{1}{\gamma }\sum_{i=1}^{N}\frac{1}{\lambda_{i}}(1-\exp(-\gamma \lambda_{i}t))u_{i}u_{i}^{\prime }y_{0}, \end{array}$$ (14)

In contrast to Φ_1_, Φ_2_(*t*) can be viewed as a function of time, but here *t* has units of years rather than milliseconds, since the model captures the slow spread of degenerating neurons. Since the true time since onset is not empirically accessible in general, we will estimate *t* as the instant *t*_*crit*_ when the theoretical pattern Φ_2_(*t*) best matches measured atrophy pattern in the subject. Both *t*_*crit*_ and the rate constant *γ* are *a priori* inaccessible, and must be empirically determined by data fitting. Seed vector **y**_0_ is known in the TLE-MTS case to a high level of confidence, since prominent hippocampal sclerosis indicates a high likelihood that it is indeed the focus location. Thus, we initialize **y**_0_ by a unit vector which is zero except for the element corresponding to the hippocampus node which is 1.

### Comparison between Model 1 and Model 2

Inspection of Eqs (10) and (14) reveals that they differ only in the indefinite integral over *t*; consequently Model 1 is simply Model 2, evaluated at *t* = ∞. Thus, one simple test of which model is better would be to determine whether the fit with real data peaks at an intermediate value of *t* (Model 2 wins) or whether the fit is monotonically increasing in *t*, peaking at *t* = ∞ (Model 1 wins).

### Study Population

The epilepsy population consisted of 29 TLE-MTS subjects (mean age 39.79±10.77, left TLE/right TLE 15/14, females/males 17/12). The presence of MTS in TLE-MTS was suggested by hippocampal subfield volumetry using high-resolution T2-weighted images aimed at the hippocampus. The TLE-no cohort consisted of fifty subjects (mean age 37.64±10.19, left TLE/right TLE 20/30, females/males 27/23). The healthy control group had mean age 35.93±9.92 and females/males 45/16. Identification of epileptogenic focus was based on seizure semiology and prolonged ictal and interictal video/EEG/telemetry (VET) in all patients. In this work non-lesional epilepsy is defined as TLE with or without MTS but no other pathology detectable on visual inspection, e.g. tumor, dysplasia, vascular or other malformation.

All imaging of epileptic subjects was performed on a Bruker MedSpec 4T system controlled by a Siemens Trio TM console and equipped with an eight-channel array coil (USA Instruments). The following sequences were acquired: (1) For cortical thickness and thalamus measurements a volumetric T1-weighted gradient echo MRI (MPRAGE): TR/TE/TI = 2300/3/950 ms, 1.0 × 1.0 × 1.0mm^3^ resolution, acquisition time 5.17min. (2) For the measurement of hippocampal subfields, a high-resolution T2-weighted fast spin echo sequence: TR/TE = 3500/19 ms, 0.4 × 0.4mm^2^ in plane resolution, 2mm slice thickness, 24 slices acquisition time of 5.30min. (3) For the determination of intracranial volume (ICV), a T2-weighted turbo spin echo sequence: TR/TE = 8390/70 ms, 0.9 × 0.9 × 3mm^3^ nominal resolution, 54 slices, acquisition time of 3.06min.

### Healthy Cohort (Connectome)

A cohort of normal subjects were collected jointly by Weill Cornell Medical College and the Brain Trauma Foundation to create the normative connectivity information in the form of tractograms, see [54] for details. Seventy three healthy subjects were used to create the normative connectivity information in the form of tractograms. T1-weighted structural and diffusion-weighted MR images were collected on a 3T GE Signa EXCITE scanner (GE Healthcare, Waukesha, WI). The High Angular Resolution Diffusion Images data were acquired with 55 isotropically distributed diffusion-encoding directions at *b* = 1000sec/mm^2 and one at *b* = 0sec/mm^2, from 72 1.8mm thick interleaved slices (no slice gap) and 128 × 128 matrix size, zero-filled during reconstruction to 256 × 256. Proposed and validated in Iturria-Medina *et al* [55], the tractography algorithm implemented here incorporates tissue classification probability and orientation distribution information using Bayesian methods.

### Analysis Outline

We define measured atrophy as the volumetrics *t*-statistics *s* obtained from a healthy group and an epileptic group (TLE-MTS and TLE-no). We assume that the neuronal atrophy resulting from epilepsy is proportional to the cortical/subcortical volumetric change relative to healthy brains. The two-sample *t*-statistic of the epileptic groups were computed relative to the healthy control group. For two samples *x* and *y* of sizes *n* and *m* and with variances $\sigma_{x}^{2}$ and $\sigma_{y}^{2}$, the *t*-statistic is given by

$$\begin{array}{c} s=\frac{\bar{x}-\bar{y}}{\sqrt{\frac{\sigma_{x}^{2}}{n}+\frac{\sigma_{y}^{2}}{m}}}, \end{array}$$

where $\bar{x}$ and $\bar{y}$ are respectively the means of *x* and *y*. In this work, *x* and *y* are the volumetrics matrices obtained from the healthy group *x* and the epileptic groups *y*. The use of the *t*-statistic between healthy and disease groups’ regional volumes in this manner is a well known and widely used surrogate for measuring atrophy at a regional level, and forms the basis for almost all brain volumetric pipelines, e.g. [26, 31, 56].

We apply the network models described by Eqs (10), (14) on the Laplacian ℒ of the 86 nodes mean structural connectivity of a population of healthy brains. In the case of the spread of activity model, Eq (10), we bilaterally seed all of the temporal nodes (vector **x**_0_ of Eq (10) has ones for the elements corresponding to both temporal lobes, and zero elsewhere). The atrophy Φ_1_ is then computed over subsets of the Laplacian eigen-modes ($\frac{1}{\beta }\sum_{i=1}^{K}\frac{1}{\lambda_{i}}{\text{u}}_{i}{\text{u}}_{i}^{\prime }{\text{x}}_{0}$, 1 ≤ *K* ≤ 86) until all eigen-modes have been exhausted. At each value of *i*, *R* between group atrophy and Φ_1_ is computed. The Φ_1_ with the highest *R* is taken as Model 1’s estimate of neuronal atrophy.

Regarding Model 2, atrophy via remote degeneration, a seed is placed at a given node, then Φ_2_(*t*) Eq (14) is computed over a range of diffusion depth values *t*. At each value of *t* the correlation of Φ_2_ with group atrophy at the given node is computed. The Φ_2_ with the highest *R* is then Model 2’s estimated atrophy for the seeded node. The process is repeated for all nodes. The Φ_2_ yielding the highest *R* of all nodes is the model’s estimate of the neuronal atrophy. In this work we discard the correlation *R* obtained in less than three time points (*t* < 3) diffusion since this does not allow the diffusion to propagate significantly into the Laplacian network. In such cases, we choose instead the highest local maximum. We note however that all of the obtained atrophy estimates result in *R* vs *t* curves with unique maxima with *t* > 2. The resulting correlation curve generally takes on a bell shape, but occasionally exhibits local maxima or even monotony, in which case we choose the time point corresponding to the highest one.

## Supporting Information

S1 Text (PDF)
