# Supplementary material for: Relating Cortical Atrophy in Temporal Lobe Epilepsy with Graph Diffusion-Based Network Models
Source: PLoS Comput Biol. 2015 Oct 29;11(10):e1004564. doi: 10.1371/journal.pcbi.1004564 (PMC4626097; doi:10.1371/journal.pcbi.1004564)
Supplement: S1 Text — (PDF) [file pcbi.1004564.s001.pdf]

Supplemental Material

<u>Computing the t-statistics between the TLE-no and TLE-MTS groups</u>:

TLE-no atrophy is known to be bilateral and broadly distributed in both cortical and subcortical regions. The atrophy due to TLE-MTS tends to spread across the hemispheres but dominates at the ipsilateral hippocampus. This can be seen in Figure A depicting the t-statistics between the volumetrics of both groups. The t-statistics remain confined within ±2std for most regions, with some regions slightly exceeding the ±2std significance threshold, the exception being the ipsilateral hippocampus, consistent with the TLE-MTS.

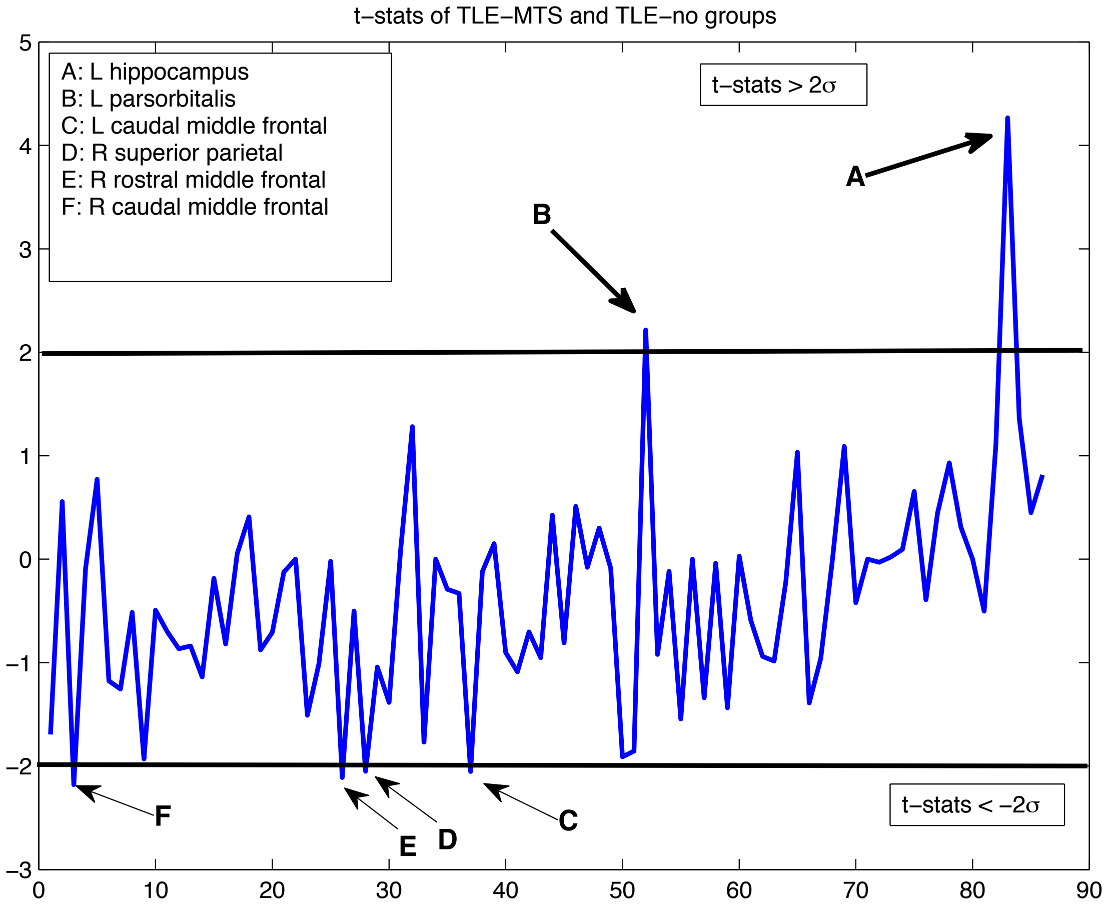

Figure A Volumetric t-statistics between the two epilepsy groups over all 86 regions. Only the ipsilateral hippocampus stands out (corresponding to TLE-MTS), as to be expected.

<u>Sensitivity of models 1 & 2 to outliers</u>:

Figure 4 in the manuscript shows the linear scatter plots of the measured neuronal atrophy vs. the estimated neuronal atrophy for both models and both types of epilepsy.

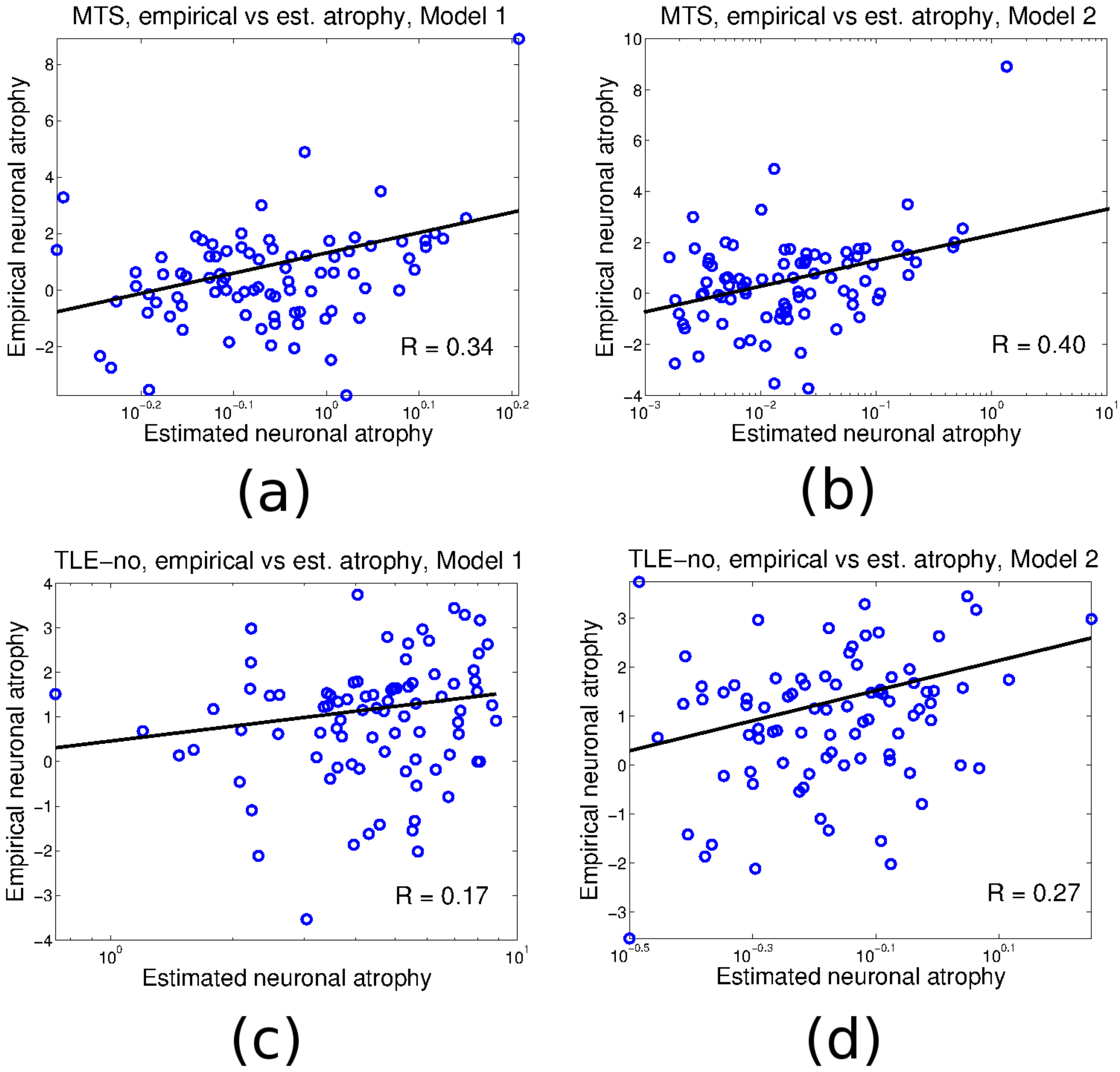

Figure B Semilogarithmic scatter plots for each model and epilepsy type of the measured neuronal atrophy vs. the neuronal atrophy as estimated by the two models (logarithmic scale). Empirical vs. estimated neuronal atrophy for the case of TLE-MTS; Model 1 (a), and Model 2 (b); and measured vs. estimated neuronal atrophy for the case of TLE-no; Model 1 (c), and Model 2 (d). For both types of epilepsy, Model 2 outperforms Model 1.

It is shown that for both types of epilepsy model 2 (progressive degenerative process) is more selective in its atrophy estimate and gives a higher Pearson correlation R with the empirical atrophy. Plotting instead the measured neuronal atrophy vs. the logarithm of the estimated neuronal atrophy can accentuate the effect of outliers. Figure B depicts the scattered plots of the empirical neuronal atrophy vs the estimated atrophy for TLE-MTS (Model 1, Fig. B(a); Model 2, Fig B(b)), and TLE-no (Model 1, Fig. B(c); Model 2, Fig. B(d)). For all four cases we compute the Pearson correlation between the empirical atrophy and the log of the estimated neuronal atrophy. As can be seen from Figure B, model 2 consistently gives higher R for both TLE-no and TLE-MTS, and it is less influenced by the outliers.
